# Coexistence of *optrA* and *fexA* in Campylobacter

**DOI:** 10.1128/mSphere.00125-21

**Published:** 2021-05-12

**Authors:** Biao Tang, Yao Wang, Yi Luo, Xue Zheng, Xiaoxia Qin, Hua Yang, Zhangqi Shen

**Affiliations:** a State Key Laboratory for Managing Biotic and Chemical Threats to the Quality and Safety of Agro-products & Institute of Agro-product Safety and Nutrition, Zhejiang Academy of Agricultural Sciences, Hangzhou, China; b Beijing Key Laboratory of Detection Technology for Animal-Derived Food Safety, Beijing Laboratory of Food Quality and Safety, College of Veterinary Medicine, China Agricultural University, Beijing, China; Antimicrobial Development Specialists, LLC

**Keywords:** *Campylobacter*, *fexA*, *optrA*, multidrug resistance

## Abstract

Previous studies indicated that Campylobacter has developed several mechanisms that confer resistance to florfenicol, which is used in food animal production. This study describes the coexistence of *optrA* and *fexA* in Campylobacter jejuni and Campylobacter coli isolates from pigs and poultry. Moreover, whole-genome sequencing data showed that the two genes are located in various multidrug resistance genomic islands within different regions of the Campylobacter genomes. The emergence of *optrA* and *fexA* may support the spread of florfenicol-resistant Campylobacter strains of animal origin.

**IMPORTANCE** Florfenicol is widely used for the treatment of respiratory infections and as a feed additive in food animal production. As a foodborne pathogen, Campylobacter is constantly exposed to florfenicol, and resistance to this antimicrobial agent has increased in recent years. Previous studies indicated that Campylobacter has developed several mechanisms that confer resistance to florfenicol. This study describes for the first time the coexistence of the florfenicol exporter FexA and the ribosomal protective protein OptrA in Campylobacter jejuni isolated from pigs. The two genes were located in various multidrug resistance genomic islands within different regions of the Campylobacter genomes. Although phenicols are not commonly used for the treatment of Campylobacter infections, the extensive use of florfenicol in food animals may play a role in the coselection of multidrug resistance genomic island (MDRGI)-carrying Campylobacter isolates which also exhibited resistance to critically important antimicrobial agents (macrolides, aminoglycosides, and tetracyclines) commonly used for the treatment of human campylobacteriosis.

## Abstract (precis)

Florfenicol is widely used for the treatment of respiratory infections and as a feed additive in food animal production. As a foodborne pathogen, Campylobacter is constantly exposed to florfenicol, and resistance to this antimicrobial agent has increased in recent years.

## OBSERVATION

Campylobacter is the leading bacterial pathogen that causes diarrheal illness worldwide, with most cases of campylobacteriosis being triggered by Campylobacter jejuni. As a foodborne pathogen, Campylobacter is constantly exposed to multiple antimicrobial agents used during food animal production. Thus, Campylobacter has developed various resistance mechanisms, including the formation of multidrug resistance genomic islands (MDRGIs), for fitness advantage upon exposure to multiple antimicrobial agents (1–4). Florfenicol is a fluorinated thiamphenicol derivative that was exclusively approved as a broad-spectrum antimicrobial agent for the treatment of animals raised for food (5). To date, several mechanisms of antibiotic resistance to florfenicol have been characterized, including the multidrug resistance protein Cfr(C), the multidrug efflux pump RE-CmeABC, and the recently described florfenicol exporter FexA and the ribosomal protective OptrA (4, 6–10). *cfr*(C), RE-*cmeABC*, and *fexA* were characterized in both C. jejuni and Campylobacter coli, whereas *optrA* was identified only in C. coli (4, 6–10).

The phenicol exporter gene *fexA* is responsible for florfenicol resistance. *optrA* not only confers resistance to phenicols but also results in elevated MICs of the oxazolidinone linezolid. Although these drugs are not commonly used for the treatment of Campylobacter infections, the extensive use of florfenicol in food animals may play a role in the coselection of MDRGI-carrying Campylobacter isolates, which also exhibit resistance to macrolides, aminoglycosides, and tetracyclines, commonly used for treating human campylobacteriosis (7). Linezolid represents one of the last-resort antimicrobial agents for the treatment of severe infections caused by methicillin-resistant Staphylococcus aureus and vancomycin-resistant *Enterococcus* spp. Thus, the coexistence of these two drug-resistant genes in Campylobacter aggravates the spread of antimicrobial resistance and poses a threat to human health. In this study, the coexistence of *optrA* and *fexA* was identified in C. jejuni isolates from pig and C. coli isolates from chicken and duck, and whole-genome sequencing was used to characterize their genetic environment.

To determine the presence of *optrA*, the primers A–F (5′-AGGTGGTCAGCGAACTAA-3′) and A–R (5′-ATCAACTGTTCCCATTCA-3′) (11) were used for PCR analysis of 146 C. coli and 54 C. jejuni strains isolated from poultry and swine farms in Zhejiang and Hunan provinces, China. The *optrA* sequence was identified in two C. jejuni and five C. coli isolates. Of note, all the strains also contained *fexA*.

To further characterize these seven *optrA*^+^ *fexA*^+^ isolates and the genetic environment of the genes, a hybrid sequencing strategy using Illumina short-read and MinION long-read technology was used to generate the complete genomes, as previously described (12). Five complete and two draft genome sequences were obtained for further mining (Table 1). *In silico* multilocus sequence typing of the whole-genome sequencing data showed that these seven isolates belonged to three sequence types (ST), including ST825, ST828, and a new ST (*aspA*_8, *glnA*_620, *gltA*_292, *glyA*_28, *pgm*_1072, *tkt*_668, and *uncA*_23). Acquired antimicrobial resistance genes can explain the resistance phenotype, including florfenicol resistance, determined by the broth microdilution method (Table 2). Only three and one single nucleotide polymorphisms were detected in the *optrA* and *fexA* sequences, respectively, in these seven strains (see Fig. S1 in the supplemental material).

**TABLE 1:** Isolation and genomic information of *optrA*^+^ *fexA*^+^ strains

Isolate	Host	Species	Genome length (bp)	GC content (%)	MLST^*a*^	Quality	MDRGI length (bp)	MDRGI GC content (%)	Accession no.
CC19DZ036	Duck	C. coli	Chromosome: 1,761,335	31.37	ST828	Completed	11,195	36.22	CP068565
CC19DZ037	Duck	C. coli	Chromosome: 1,761,334	31.37	ST828	Completed	11,195	36.22	CP068566
CC19PF050	Pig	C. jejuni	1,798,069	30.21	Unknown	Draft			JAESVI000000000
CC19PF065	Pig	C. jejuni	Chromosome: 1,681,082	30.57	Unknown	Completed	18,223	34.79	CP068567
			pPF065-186: 186,647	26.41					CP068568
			pPF065-3: 3,395	31.37					CP068569
CC19CH074	Chicken	C. coli	1,831,137	31.16	ST825	Draft			JAESVJ000000000
CC19CH075	Chicken	C. coli	Chromosome: 1,781,472	31.47	ST825	Completed	18,553	36.36	CP068581
			pCH075-80: 80,135	26.07					CP068582
			pCH075-4: 4,944	29.57					CP068583
			pCH075-3: 3,405	31.01					CP068584
			pCH075-2: 2,426	25.89					CP068585
CC19CH076	Chicken	C. coli	Chromosome: 1,781,471	31.47	ST825	Completed	18,553	36.36	CP068586
			pCH076-80: 80,131	26.09					CP068587
			pCH076-4: 4,944	29.57					CP068588
			pCH076-3: 3,405	31.01					CP068589
			pCH076-2: 2,426	25.89					CP068590
ZS007	Duck meat	C. jejuni	Chromosome: 1,658,567	30.55	ST10317	Completed	22,697	38.01	CP048771
1712SZ1KX20C	Chicken	C. coli	1,713,884	31.36	ST825	Draft	9,611	36.79	JAATKE000000000

a MLST, multilocus sequence type.

**TABLE 2:** Acquired drug resistance genes and MIC of *optrA*^+^ *fexA*^+^ isolates

Isolate	Acquired drug resistance genes	MIC (μg/ml) of^*a*^:								
		CIP	NAL	GEN	TET	CLI	ERY	AZM	TEL	FFC
CC19DZ036	*tet*(L), *tet*(O), *optrA*, *fexA*, *cat*A9, *bla*_OXA-61_	16	64	0.5	32	4	>64	>32	32	>32
CC19DZ037	*tet*(L), *tet*(O), *optrA*, *fexA*, *cat*A9, *bla*_OXA-61_	16	128	0.5	32	4	>64	>32	32	>32
CC19PF050	*aac*(6′)-*aph*(2′′), *aph*(3′)-III, *aph*(2′′)-If, *ant*(6)-Ia, *tet*(L), *tet*(O), *optrA*, *fexA*, *cat*, *cat*A9, *bla*_OXA-465_, *erm*(B)	16	>128	>64	>64	>32	>64	>32	32	>32
CC19PF065	*aac*(6′)-*aph*(2′′), *aph*(3′)-III, *aph*(2′′)-If, *ant*(6)-Ia, *tet*(L), *tet*(O), *optrA*, *fexA*, *cat*, *cat*A9, *bla*_OXA-465_, *erm*(B)	16	128	>64	>64	16	>64	>32	32	>32
CC19CH074	*aac*(6′)-*aph*(2′′), *aph*(3′)-III, *ant*(6)-Ia, *tet*(L), *tet*(O), *optrA*, *fexA*, *cat*, *cat*A9, *bla*_OXA-61_, *erm*(B)	32	64	>64	64	>32	64	32	32	>32
CC19CH075	*aac*(6′)-*aph*(2′′), *aph*(3′)-III, *ant*(6)-Ia, *tet*(L), *tet*(O), *optrA*, *fexA*, *cat*, *cat*A9, *bla*_OXA-61_, *erm*(B)	32	64	>64	64	>32	64	16	16	>32
CC19CH076	*aac*(6′)-*aph*(2′′), *aph*(3′)-III, *ant*(6)-Ia, *tet*(L), *tet*(O), *optrA*, *fexA*, *cat*, *cat*A9, *bla*_OXA-61_, *erm*(B)	32	64	64	64	>32	>64	32	16	>32

a Abbreviations: CIP, ciprofloxacin; NAL, nalidixic acid; GEN, gentamicin; TET, tetracycline; CLI, clindamycin; ERY, erythromycin; AZM, azithromycin; TEL, telithromycin; FFC, florfenicol. CLSI- or NARMS-approved breakpoint concentrations for resistance, in micrograms per milliliter, are as follows: CIP, 4; NAL, 32; GEN, 4; TET, 16; CLI, 8; ERY, 32; AZM, 1; TEL, 8; FFC, 8.

10.1128/mSphere.00125-21.1 FIG S1 Comparison of nucleic acid homology of *fexA* and *optrA* genes in different strains. Download FIG S1, DOCX file, 0.3 MB. Copyright © 2021 Tang et al. 2021 Tang et al. https://creativecommons.org/licenses/by/4.0/ This content is distributed under the terms of the Creative Commons Attribution 4.0 International license.

**FIG 1:**
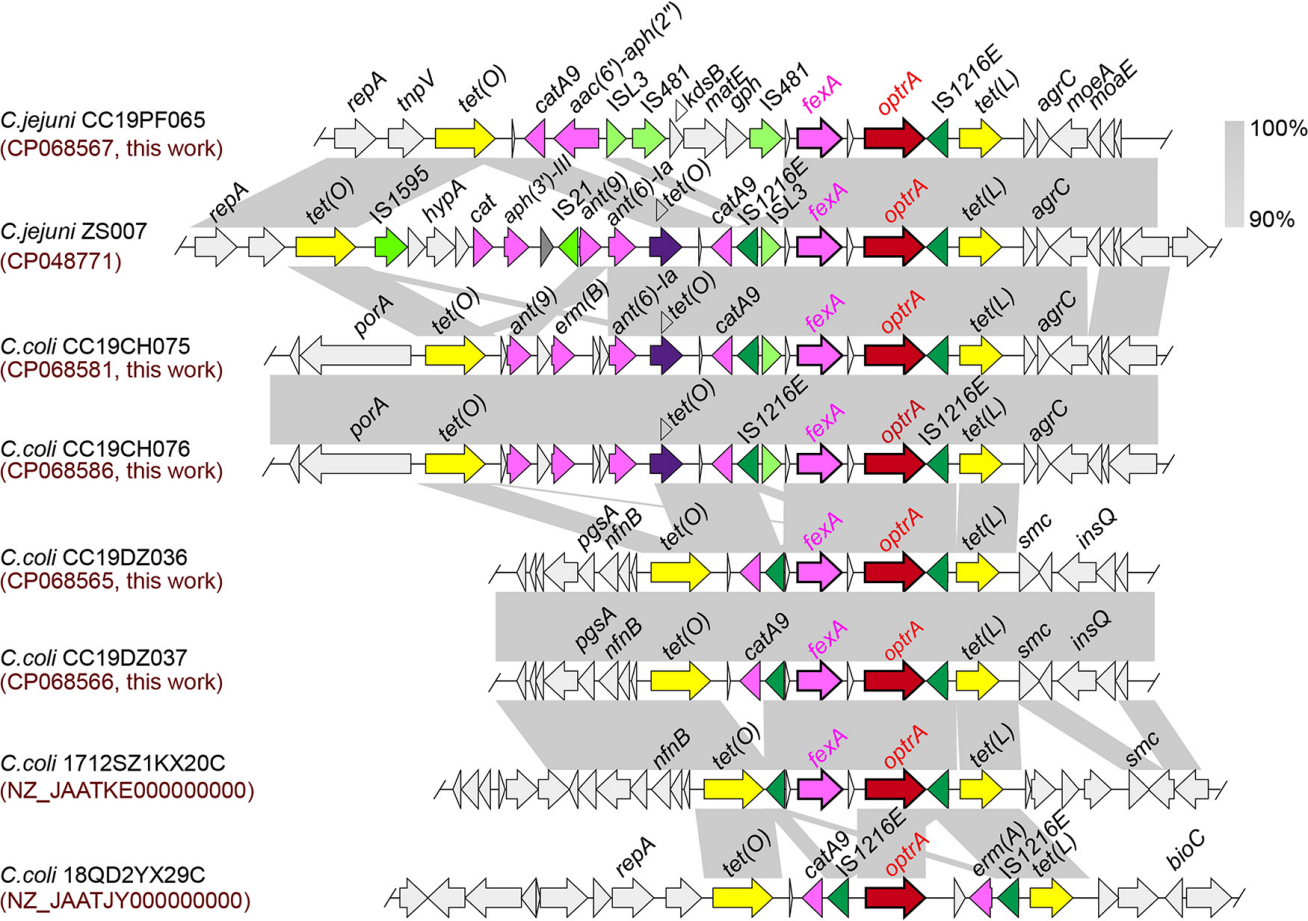
Genetic environment of *optrA* in the genomes of C. jejuni isolates and comparison of the *optrA*-carrying regions. Arrows indicate the transcription direction. Regions of >90% homology are marked with gray shading. Genes are differentiated by different colors.

C. jejuni CC19PF065 belongs to a new ST (the nearest STs are 8670, 8672, and 10317), and the complete genome was 1,681,082 bp in length, with a GC content of 30.57% (accession no. CP068567). *optrA* along with its upstream phenicol exporter *fexA* and the gene *hp*, which encodes a hypothetical protein, were located within a 18,223-bp MDRGI with 34.79% GC content. The formed the MDRGI *tet*(O)*-hp-catA9-aac*(6′)*-aph*(2′)-IS*L3-*IS*481-ΔkdsB-matE-gph-*IS*481-hp-fexA-hp-optrA-*IS*1216E-tet*(L) (Fig. 1) was inserted into the C. jejuni housekeeping genes, between *repA* and *agrC*. In addition to *fexA* and *optrA*, this MDRGI also contained other genes conferring resistance to tetracycline and aminoglycosides. Moreover, *ΔkdsB* (3-deoxy-manno-octulosonate cytidylyltransferase), *matE* (multiantimicrobial extrusion protein), and *gph* (phosphoglycolate phosphatase) were flanked by the insertion sequence IS*481* in the same orientation. The segment IS*481-ΔkdsB-matE-gph-*IS*481* (3,709 bp; 36.88% GC content) exhibited 78.3% nucleotide sequence identity to the corresponding region of Helicobacter cholecystus strain NCTC 13205 (accession no. LR134518), with *matE* encoding a novel MATE family efflux transporter, most likely from *Helicobacter* (Fig. S2).

10.1128/mSphere.00125-21.2 FIG S2 (A) Comparative analysis of *ΔkdsB*-*matE*-*gph* regions between strain Z65 CC19PF065 and Helicobacter cholecystus NCTC 13205. (B) Phylogenetic analysis of MatE protein of C. jejuni Z65 CC19PF065. Download FIG S2, DOCX file, 0.3 MB. Copyright © 2021 Tang et al. 2021 Tang et al. https://creativecommons.org/licenses/by/4.0/ This content is distributed under the terms of the Creative Commons Attribution 4.0 International license.

C. jejuni ZS007 belonged to ST10317, with a complete genome 1,658,567 bp in length and a GC content of 30.55% (accession no. CP048771). This strain was previously reported to contain *fexA*. Here, the *optrA* containing MDRGI was found to be 22,697 bp in length (38.01% GC content) (Table 1). The order of gene content was *tet*(O)-IS*1595-hp-hypA-hp-cat-aph(3′)-III-hp-*IS*21-ant*(9)*-ant*(6)*-Ia-Δtet*(O)*-hp-catA9-*IS*1216E-*IS*L3-hp-fexA-hp-optrA-*IS*1216E-tet*(L), which was inserted between *repA* and *agrC*. The fragment *hp-fexA-hp-optrA-*IS*1216E-tet*(L) was the same as that from C. jejuni CC19PF065. The segment IS*1595-hp-hypA-hp-cat-aph(3′)-III-hp-*IS*21* showed 98.92% nucleotide sequence identity to the corresponding region of the C. jejuni strain BC chromosome (accession no. CP032522).

In addition, a novel *optrA*-containing MDRGI was also characterized in C. coli by comparing the results from published studies (7, 9). According to the sequencing results, C. coli CC19CH075 and CC19CH076 belonged to ST825, and the complete genome sequences were 1,781,472 bp (accession no. CP068581) and 1,781,471 bp (accession no. CP068586) in length, with a GC content of 31.47% (Table 1). In total, 60 single nucleotide polymorphisms and seven gaps were found between the two strains. The order of gene content was *tet*(O)*-hp-ant*(9)*-hp-erm*(B)*-hp-hp-ant*(6)*-Ia-Δtet*(O)*-hp-catA9-*IS*1216E-*IS*L3-hp-fexA-hp-optrA-*IS*1216E-tet*(L), which was inserted between *porA* and *agrC.* By comparing with the segment from C. jejuni ZS007, *hp-ant*(9)*-hp-erm*(B)*-hp-hp* was found to be replaced by IS*1595-hp-hypA-hp-cat-aph*(*3′)-III-hp-*IS*21-ant*(9) in C. coli strains CC19CH075 and CC19CH076.

C. coli CC19DZ036 and CC19DZ037 belong to ST828, and the complete genomes were 1,761,335 and 1,761,334 bp in length, with a GC content of 31.37% (Table 1) (accession no. CP068565 and CP068566, respectively). In total, only three gaps difference were found between the two strains. The order of gene content was *tet*(O)*-hp-catA9-*IS*1216E-hp-fexA-hp-optrA-*IS*1216E-tet*(L), which was inserted between *nfnB* and *smc*.

This study revealed that the emerging gene *optrA* is associated with various MDRGIs in C. jejuni. Moreover, the core segment *fexA-hp-optrA-*IS*1216E* was identified in both C. jejuni and C. coli, which agrees with previous reports (7). Of note, the *optrA-*containing MDRGIs varied from 9,611 to 22,697 bp and were inserted into different regions over the genomes of Campylobacter, all of which contained *tet*(O) and *tet*(L) at the two ends. The GC content of these MDRGIs ranged from 34.79% to 38.01%, which is different from the GC content of the Campylobacter genome (∼31.0%), suggesting that Campylobacter might have obtained these MDRGIs from other species. There were 12 antimicrobial resistance genes in MDRGI, including *aac*(6′)-*aph*(2′′), *aph*(3′)-III, *aph*(2′′)-If, *ant*(6)-Ia, *tet*(L), *tet*(O), *optrA*, *fexA*, *cat*, *catA9*, *bla*_OXA-465_, and *erm*(B), which were resistant to aminoglycosides, tetracyclines, phenicol, and macrolides. All of these antibiotics were used for the prevention and treatment of infections in farm animals in China. In addition, the GC content within several MDRGIs was not evenly distributed, and the presence of multiple insertion sequences suggested that their integration may have occurred through a multistep process. Therefore, MDRGI in Campylobacter was likely to be the product of multiple-antibiotic coselection. Due to the use of florfenicol in livestock and poultry production, the emergence of *fexA* and *optrA* could confer a fitness advantage under selection pressure, which will support the spread of *fexA* and *optrA* and their associated MDRGIs through Campylobacter natural transformation.
