# Supplementary figures and images for: Coexistence of optrA and fexA in Campylobacter
Source: mSphere. 2021 May 12;6(3):e00125-21. doi: 10.1128/mSphere.00125-21 (PMC8125047; doi:10.1128/mSphere.00125-21)

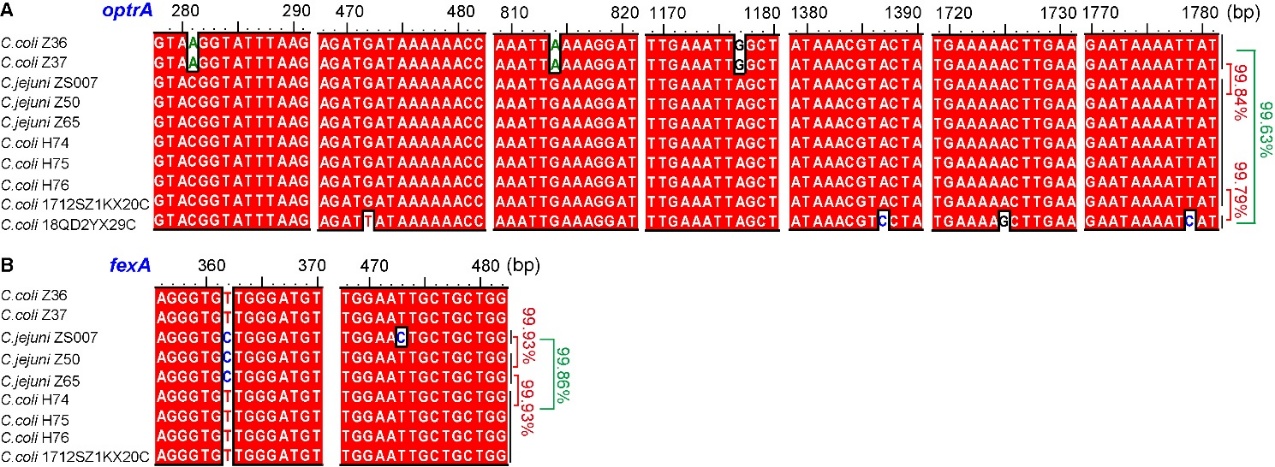

Supplement: FIG S1 [file mSphere.00125-21-sf001.docx]

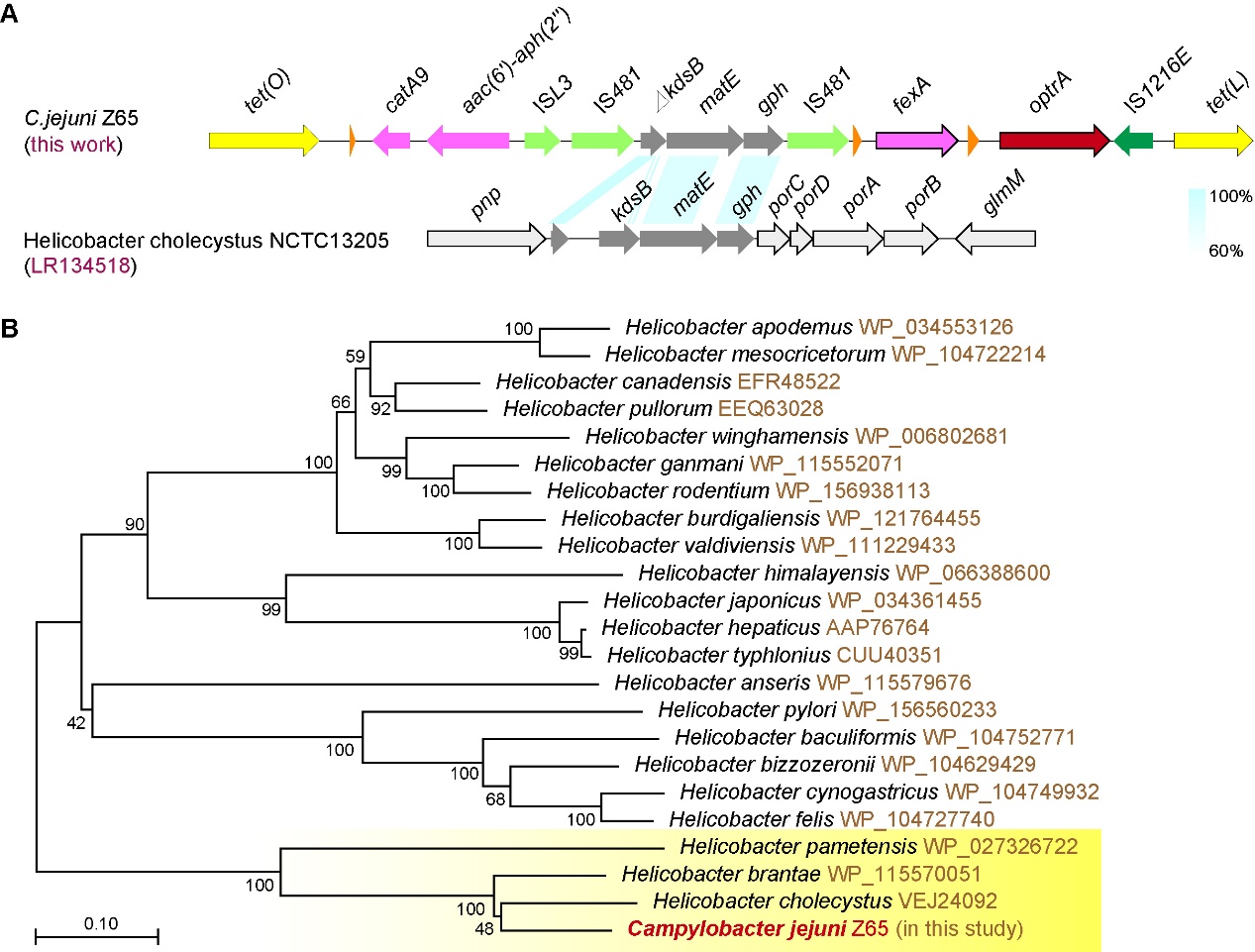

Supplement: FIG S2 [file mSphere.00125-21-sf002.docx]
